# Sustainable urban mobility analysis for elderly and disabled people in São Paulo

**DOI:** 10.1038/s41598-020-80906-w

**Published:** 2021-01-12

**Authors:** Gislaine A. Azevedo, Renelson R. Sampaio, Aloisio S. Nascimento Filho, Marcelo A. Moret, Thiago B. Murari

**Affiliations:** 1 SENAI CIMATEC University Center, Salvador, Brazil; 2 University of the State of Bahia (Universidade do Estado da Bahia, UNEB), Salvador, Brazil

**Keywords:** Applied physics, Statistical physics

## Abstract

The Brazilian Urban Mobility Policy integrates urban transport with traffic planning, establishing appropriate public policies that indicate the need for a safe and accessible public transport system. The major challenge is the inclusion of the elderly and people with disabilities. In this paper, we quantify the impact of rainfall on the number of people with disabilities and elderly people who use the public bus transportation system for accessibility in the first and last miles in the city of São Paulo. The proposed methodology is used to evaluate the co-movements between the time series of free-fare users and rainfall in São Paulo. The findings confirm the hypothesis that significant rainfall causes a reduction in the number of daily free-fare passengers who use the public bus system in São Paulo.

## Introduction

The population of urban areas, notably in the main metropolises, has continuously grown worldwide^1^. Brazil comprises 63 cities with populations above 300,000 inhabitants^2^. The city of São Paulo, with around 12.8 million inhabitants, stands out due to its strong urbanization over the last decades^3^, registering an average rate of 0.80% in the period of 2010–2020. This growth directly affects the circulation of its population in urban areas, including via transportation systems^4^. However, a gradual reduction in its growth is expected, with an annual rate of − 0.09% between 2040 and 2050^5^.

Models, techniques and methods are constantly applied in scientific research for urban planning and development^6^, seeking solutions that improve urban mobility and mitigate the most pressing problems (e.g. urban isolation, social injustice and inaccessibility to opportunities)^1^. Public transport, including the first and last mile stages, that is the short distance between the home and public transport or between public transport stations and the workplace that are known as the first and last miles^7^, is a crucial part of the urban environment. There is a constant need for mobility solutions that provide an efficient infrastructure that fulfills movements and includes the population in the urban system^6^. Although urban mobility has been addressed in several studies, a gap in the literature is the lack of social inclusiveness regarding accessibility for all individuals^4^. The life expectancy of the population has increased because of the constant transformation, growth, and progress of society. This means the number of elderly people^8^ and persons with disabilities (PWD) has increased^9^.

The percentage of elderly people over 60 years old is estimated to reach 22% of the world population by 2050^8^, while the estimated number of PWD for 2050 is 6.25 billion^9^. These are significant growths in these populations considering the current numbers. Accessibility plays an important role in society and is one of the greatest challenges faced in large cities^10^. For instance, the mobility of PWD and elderly people is hampered due the vast, complex, and highly dynamic system in the urban environment^11^.

Accessibility is lacking in the connections between different modes of transport for these groups of people, mainly in the first and last miles of the journey. The journey and access to public bus transport can be considered prominent problems. They are related to a lack of accessibility and the non-inclusion of PWD and elderly people^12^. The first and last miles can be traveled by motor vehicles, such as cars, scooters, motorcycles, and bicycles, or even on foot. However, little attention has been paid to the availability and accessibility of these modes of transportation for PWD and the elderly^7^.

Urban policies are fundamental to ensuring the sustainable and reliable movement of goods and people, especially in transportation and urban space planning^13^. In Brazil, the Urban Mobility Policy seeks to integrate urban transport and traffic planning, establishing appropriate public policies that indicate the need for a safe and accessible public transport system^14^. The major challenge is guaranteeing mobility for PWD^15, 16^.

Accessibility measurement has been explored in several studies^17^; however, a gap exists regarding the analysis of the possibility of accessibility being hampered by external factors. Meteorological conditions can impact public transport systems in various ways and have the potential to reduce the number of transported passengers^18^. Rain is one of the weather conditions that directly affects everyone, especially PWD and elderly people. The accessibility to public transport systems can be affected by variations in weather, which should be considered in analyses of short- or long-term time series^17^. PWD and elderly people are strongly affected by those conditions when attempting to reach public bus transport.

The mobility of the elderly is a crucial element in overall life satisfaction and active ageing as it ensures independence, good health, and quality of life^19–23^. Typically, mobility, which refers to a person’s ability to move in an independent and safe manner from one place to another, declines gradually as people age^24^. At this age, functional impairment will be more frequent^25^. Therefore, mobility should be regarded as an important part of promoting overall societal development and provide political and economic motivation for including and supporting this group of people, especially in the transport sector^26, 27^.

PWD have long been placed in a condition of incapacity due to environmental factors and body limitations, which are not recognized as a circumstance of health impairment, placing these people in a segregated position without solutions^28^. In 2006, the United Nations (UN) Convention on the Rights of Persons with Disabilities proposed some initiatives to guarantee equality based on the human rights of PWD^9^. To guide the consideration of the rights of PWD, the global report on disability includes this issue in all areas of interaction in society^9^.

In Brazil, the term PWD is defined by Decree no. 6949 of 25 August 2009, promulgated in Art. 1 of the Convention on the Rights of Persons with Disabilities, as people who face long-term difficulties of a physical, mental, intellectual, and/or sensory nature that can fully or partly restrict their participation in society due to various barriers under equitable conditions to other people^10^.

The issue of disability is not trivial; it is a complex, dynamic, multidimensional, and contested subject^29^. Transforming this perspective changes the PWD model from a view of handicap of the individual physical ability to social failure. This change in the understanding of disability transfers the paradigm from a medical to a social nature. This discussion conducted by scientists in the field of health and social sciences recognizes the problem of disability as a social and physical barrier of cities and not of the individual^30–32^. It is necessary to weigh the approach to disability, as it can be experienced because of problems in physical condition, which differs from a solely medical point of view. Therefore, care must be taken to create an approach that is not exclusively social or exclusively medical^33–35^.

A household survey conducted between 2002 and 2004 by the World Health Organization (WHO) provided a global estimate of the prevalence of disability. The questionnaire covered the health of individuals in several domains^36^. The survey was conducted in approximately 70 countries, of which 59 countries with data were discussed in the study because they represented 64% of the world population. The countries in the study were chosen based on several considerations^36^:

- The need to fill data gaps in geographic regions where data were mostly absent, such as in sub-Saharan Africa;
- Inclusion of countries with high, medium, and low income, with a focus on low- and middle-income countries;
- Inclusion of countries with large adult populations.

The questions on functional difficulties were answered with the following options: no difficulty, mild difficulty, moderate difficulty, severe difficulty, and extreme difficulty, with a calculated score composed of incapacity between 0 and 100, where 0 represented the absence of disability, and 100 represented complete disability. However, a cut-off score of 40 on a scale of 0 to 100 was defined to include individuals who faced significant difficulties in the disability estimates, and a cut-off score of 50 was established to estimate the prevalence of people with significant difficulties^9^.

This survey provided some results relevant to the present study, for instance, the mean prevalence rate of disability was 15.6% (approximately 650 million of the estimated individuals and 4.2 billion adults aged 18 years or older in 2004) for the 59 countries surveyed, with 11.8% in high-income countries and 18.0% in low-income countries. The average prevalence rate for adults with significant difficulties was estimated to be 2.2% or approximately 92 million people in 2004^9^. If the prevalence numbers are extrapolated to cover adults aged 15 years or older, approximately 720 million people have mobility difficulties and approximately 100 million have significant difficulties. The prevalence of disability in low-income countries among people aged 60 years or older was 43.4% compared with 29.5% in high-income countries^9^.

Although this household survey conducted by the WHO is relevant regarding the number of people with mobility difficulties, the medical model of disability, which is illness-based, is used to define disabled people and the problem they face in the survey^29, 37^. The social model of disability was coined in the 1980’s by Mike Oliver^38^ to shift the focus from the body as the originator of the problem to the social barriers and societal actions that limit and exclude people with disabilities^39, 40^. However, the social model is still often not applied for identifying and eradicating disabling barriers such as in the case of the study of accessibility to public bus transportation of people with disabilities and the elderly in the first and last miles in São Paulo.

In alignment with some of the Sustainable Development Goals (SDGs), goal number 10 (Reduce inequalities) requires promotion of the social, economic, and political inclusion of people of all ages, disabilities or any other status, and goal number 11 (Sustainable cities and communities) encourages the creation of the most inclusive, safe, resilient, and sustainable cities and communities. These are goals for 2030. Sustainable mobility and transport networks reinforce this SDG^41^ through inclusive urban planning that considers the expected population growth^42^ and is resilient against climate change and extreme weather events^43^. Identifying the regions of the city that need action regarding the infrastructure of streets and public transport is important to ensure the inclusion of all citizens. This is most evident in developing countries where the urban poor rely heavily on public transport for commuting^44^ and where 80% of persons with disabilities live^45^.

As the elderly population is growing significantly in almost every country in the world, this transformation necessitates adaptation in almost all sectors involving goods or services, such as housing, transportation, and social protection; labor and financial markets; and family structures and intergenerational ties^46^. To understand how the elderly choose to travel in the city of São Paulo, a study was conducted using indices calculated with the number of trips made using a certain method, divided by the total value of trips in each traffic zone^47^. In 58% of the 55,500 trips analyzed, the elderly used private means of transport (car, motorcycle, and taxi); however, 21% traveled by public transport, 16% by bus, 5% by subway and train, and 21% on foot. Therefore, 42% of elderly trips in the city of São Paulo are partially or totally on foot^47^.

The five reasons why the elderly chose a certain type of travel were calculated based on the number of trips for a given reason divided by the total value of trips for each transport type in the area. The survey indicated that 44% of them use transport to go to work, to the doctor and study^47^. Cities must prepare for the growth of the elderly population and plan actions to meet the goals of the 2030 Agenda for Sustainable Development. The issues that arise with population ageing are relevant to the SDGs, as they are directly linked to the reduction of inequality within and between countries and to the promotion of peaceful and inclusive growth, employment, sustainable human settlements, gender equality, poverty eradication and health^46^.

Every year, planning and public policies directed toward elderly people are needed, as the population is ageing more and experiences various disabilities, and has needs regarding medical care, infrastructure, social protection, housing, and employment, among others^46^.

Several studies have reported the potential impacts of climate change on all types of natural and social systems over the last two decades^48^. The 21st century has provided an indication of the expected severity of climatic change^49^. Potentially, all natural and human systems are at risk of being affected by changes caused by a new climate regime^50^. Extreme and adverse weather conditions influence the performance of transport systems in general. However, more populated areas experience the most severe impact on transport systems because a chain reaction can be triggered by a single weather event^51^.

A unique event on 18 January 2007 in the United Kingdom, called “Windy Thursday”, has become an example of the impact of weather conditions on transport. The strong winds overturned almost 50 vehicles transporting goods, causing damage estimated at GBP 50 million^52^. The operational efficiency of the modes of transportation, such as roads, railways, and water transport, is constantly subject to meteorological hazards^53^ that can cause various harms to users, including injury and death. For these reasons, it is important to consider weather effects to ensure the means of transport of goods and people are as efficient as possible^54^. These problems can be caused by wind, as experienced on Windy Thursday^55^, as well as rain^56, 57^, high temperatures^58, 59^, ice, and snow^60^.

The Climate Impacts Program and the Intergovernmental Panel on Climate Change are climate-change-related organizations in the United Kingdom that consider weather effects when designing new transport or adapting current transport modes^50, 61^. It is important to evaluate the impact of weather conditions on the transport system; some studies have highlighted the diversity of impacts on various transport modes. Empirical statistical models or process-based models can contribute to the assessment of the impact of weather on the transport area, thus establishing the relationship between the magnitude of a given meteorological pattern and the interruption rates caused in transport^62^.

The prediction of the impact of climatic change on transport modes began in the United Kingdom with some projects aiming to stabilize the effects of these impacts. These projects, such as BIONICS (Biological and Engineering Impacts of Climate Change on Slopes) implemented under the SKCC (Sustaining Knowledge for a Changing Climate), focused on existing structure, but they did not consider socioeconomic changes. For instance, interruptions in the transport sector due to meteorological conditions may generate economic impacts^52^. The possibility of predictability is limited to a set of variables, which hinder future understanding. However, some scenarios that may be presented are able to increase understanding of the impact of climatic changes that are contributing to socioeconomic factors affecting the availability of a transport system vulnerable to climatic effects^52^.

In the present study, we aimed to quantify the impact of rainfall on the number of PWD and elderly people who use the public bus transportation system for transport in the first and last miles in São Paulo. Our hypothesis was that weather conditions, such as rain, cause a reduction in the number of PWD and elderly people that use the public bus transport in the city. This proposition has significant social, political, and economic relevance. This subject is considered pertinent by the organizing committee of the UN Agency for Cities (UN-Habitat). The sustainable mobility campaign aims to create more inclusive cities, expanding public transport to PWD and elderly people to increase mobility and accessibility in and between cities^63–65^. The originality of this study lies in the use of the cross-correlation method through the DCCA coefficient. The use of this method to analyze the impact on accessibility on the first and last miles in an urban city has not been found in the existing literature.

This paper is divided into four parts. The results are presented in Section 2. Section 3 provides a discussion concerning the results. The last section describes the methods, outlining the statistical method applied to determine the correlation by analyzing the co-movements between the time series.

## Results

**Figure 1:**
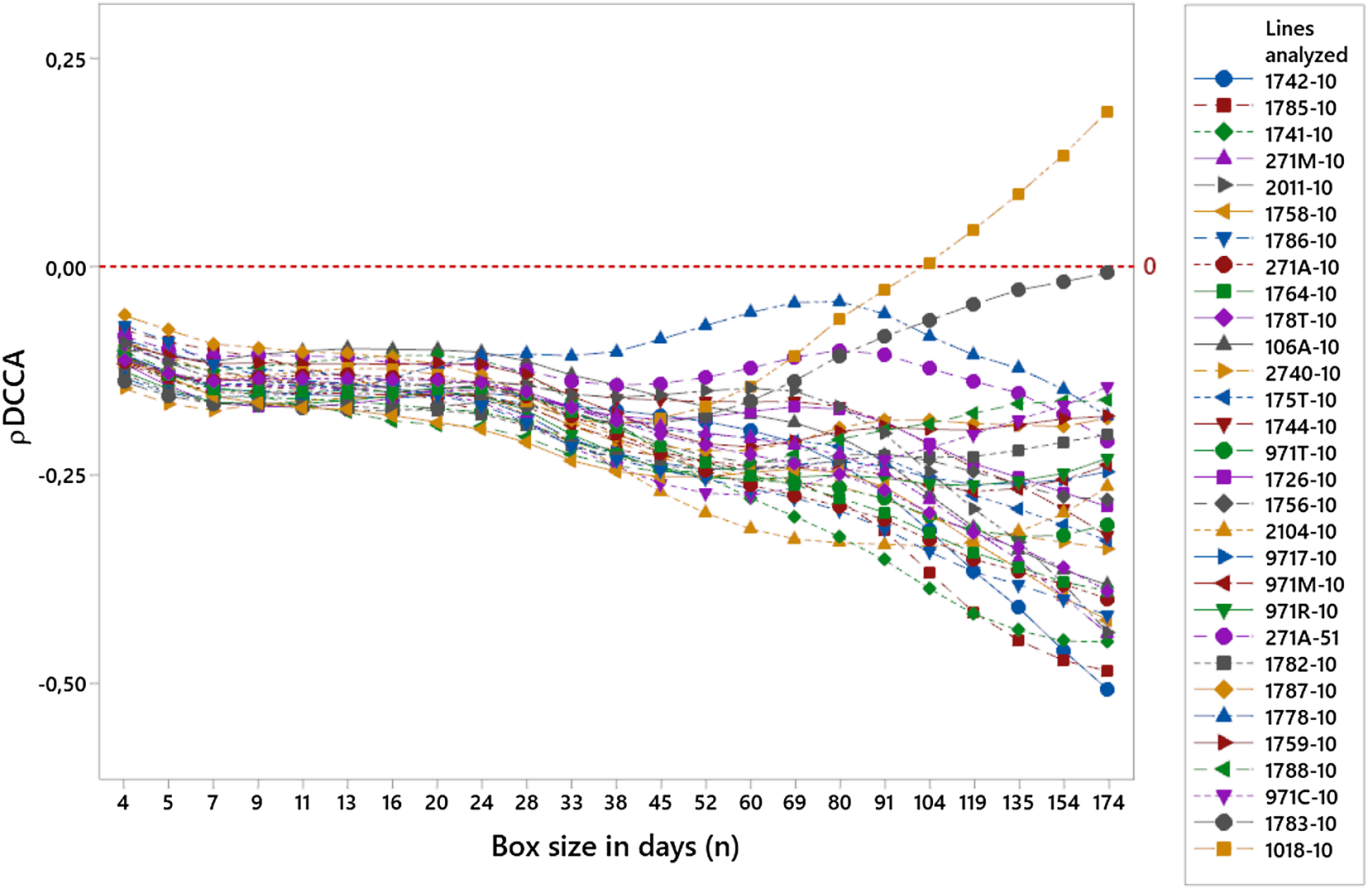
Cross-correlation between the number of people with disabilities (PWD) and elderly passengers riding the public bus system (in the 30 lines analyzed) and rainfall (mm) in the city of São Paulo, Brazil, from January 2015 to 2017.

In this section, we analyze the effects of the cross-correlation between the data series of PWD and elderly passengers riding public buses and rainfall in millimeters. In 93% of the evaluated bus lines, a negative trend was observed for boxes (*n*) of 24 days or more, as shown in Fig. 1. This change in trend starts within the macroweather regime, for which the inner scale starts at approximately 10 days^66^. The assessment of the correlation behavior should be considered from 24 days onwards when the beginning of a trend in the curves is observed and therefore the most appropriate results are considered to perform an analysis according to the purpose of this study.

The values found in 28 of the 30 bus lines analyzed using the $\rho DCCA$ coefficient were between − 0.50843 and − 0.14394 for boxes of 174 days. Therefore, all of these values for the 28 bus lines are below the red dashed line at the point at which the $\rho DCCA$ coefficient is equal to zero, which reveals a cross anti-correlation of all these bus lines. However, two bus lines (lines 1018-10 and 1783-10) showed the opposite behavior. For line 1018-10, which showed a positive cross-correlation in a box (*n*) of 174 days, the result showed that the number of PWD and elderly passengers that ride this line increases with rainfall. The cross-correlation coefficient for line 1783-10 is closer to zero, suggesting that precipitation does not affect the use of the line by the elderly and PWD passengers.

**Figure 2:**
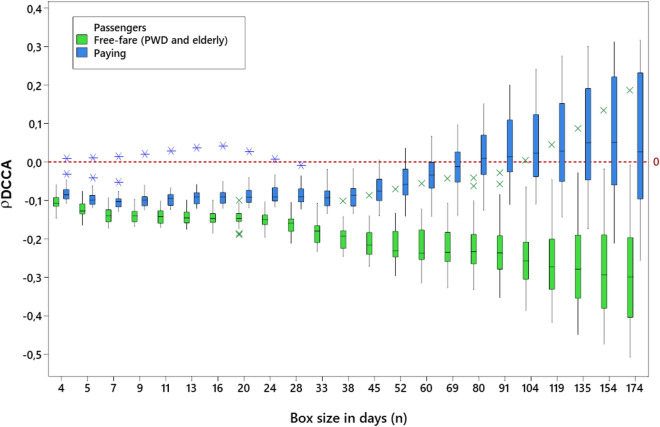
Boxplot with a correlation trend between PWD and elderly passengers (free fare) $\times$ other passengers (paying).

The behavior of PWD and elderly passengers was validated based on comparison with the group of passengers without reduced mobility, i.e., paying passengers. As shown in Fig. 2, the medians in the boxplot show a trend toward positive values and values above zero for a box (*n*) are equal to 80 days for the group of paying passengers. However, the opposite was observed in the behavior of the medians for free-fare passengers (PWD and elderly people), with negative values for all evaluated scales and a negative trend after 24 days.

Figure 2 shows that the curves are statistically different. There is no overlap of the second and third quartiles for boxes from 7 days onwards. This condition remains up to the end of the scale. The distance between the medians of the coefficients increased with the increase in the size of the boxes. The boxplot revealed that the bus line 1018-10 is an outlier. Further work needs to be completed to understand the cause of the behavior found for this bus line. Thus, the presented results confirm the hypothesis that an increase in the rainfall decreases the numbers of PWD and elderly passengers who use public buses in the city of São Paulo, as 93.3% of the analyzed bus lines presented a long-range negative cross-correlation coefficient.

We identified the region most impacted by the rainfall in the evaluated area. Three of the seven bus lines with cross-correlation values below − 0.4 are located in the area closer to the Cachoeirinha neighborhood, located on the outskirts of the northern zone of São Paulo. The average salary in Cachoeirinha is only one-fifth of the average salary in the wealthiest neighborhood. We used Google Maps to evaluate bus line 1742-10, which presented the worst cross-correlation coefficient regarding free-fare passengers. Of the 57 bus stops, 30% have a roof and seat for passengers waiting for the arrival of the bus and we did not identify suitable sidewalks for pedestrian traffic for a significant part of the route. In addition, this route has many natural obstacles, like road slopes.

**Figure 3:**
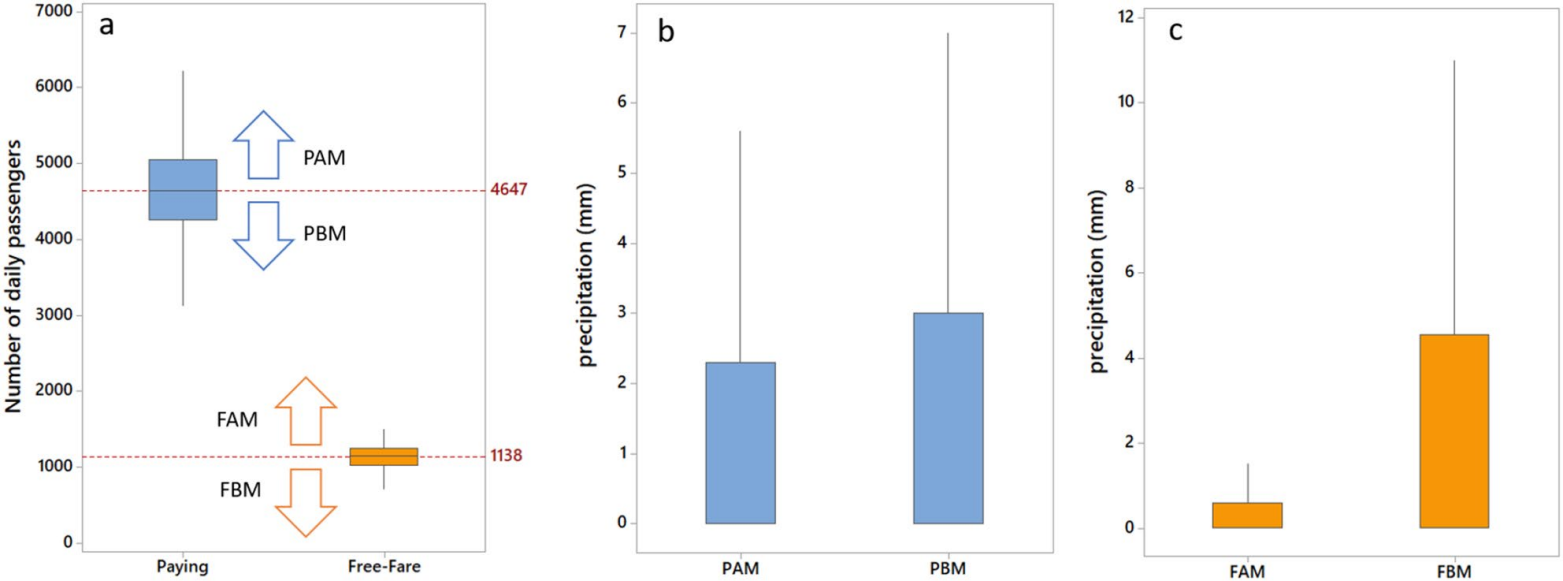
Boxplot of the number of daily paying and free-fare passengers for bus line 1742-10. Each group was split into sub-groups below and above the median line (**a**). The precipitation is not significantly visually different for paying above median line (PAM) and paying below median line (PBM) passengers (**b**), but there is a significant difference between free-fare above median line (FAM) and free-fare below median line (FBM) passengers (**c**).

The relationship between the increase in precipitation and the decrease in free fares for line 1742-10 can be visually evaluated in Fig. 3. We split paying and free-fare passengers below and above the median line for the number of daily transport accesses (Fig. 3a). There are no visually significant differences between the precipitation for paying above median line (PAM) and paying below median line (PBM) (Fig. 3b). However, precipitation for free-fare above median line (FAM) passengers is below 2 mm, but the precipitation for free-fare below median line (FBM) passengers is higher than 11 mm, without outliers (Fig. 3c), demonstrating that the number of free-fare passengers increases when rainfall is not significant.

In contrast, the bus line 1783-10 can be identified in Fig. 1 as minimally impacted by the rainfall because the $\rho DCCA$ coefficient is closer to zero for the long-range condition; 40% of its bus stops have roofs and seats. We also identified suitable sidewalks, with fewer obstacles for pedestrian traffic along the part of the route that is most densely populated. This route also has fewer road slopes than the 1742-10 line.

## Discussion

The empirical results confirm the hypothesis that significant rainfall causes a reduction in the number of daily PWD and elderly passengers who use the public bus system in the city of São Paulo. Of the 30 bus lines analyzed, 28 presented a $\rho DCCA$ coefficient below zero for long-range boxes, which revealed a negative cross-correlation of all these series. An increase in precipitation causes a reduction in the numbers of PWD and elderly passengers on several bus lines and various city regions, with different impacts on the number of these passengers who use the public bus system caused by the rainfall.

The $\rho DCCA$ coefficient allowed the identification of the worst bus lines and city areas in respect of the accessibility of the free-fare group during the rainy season in the city. The $\rho DCCA$ coefficient scale can be used to quantify mobility issues in the first and last mile trip of elderly. The closer the coefficient is to –1, there is a significant reduction in PWD and elderly people who decide to use this type of transport.

The results of this study show that measures are needed to create cities in alignment with the SDGs and to have discussions that do not simply see disabled people as the problem but acknowledges that societal barriers and actions limit and exclude people with disabilities^39^. Applying the social model to the discussions helps to reduce inequalities experienced by people with disabilities in discussions around sustainable mobility and transport networks within urban planning that considers expected population growth and resilience to climate change and extreme weather events.

The $\rho DCCA$ cross-correlation coefficient enabled the identification of areas in the city where passengers with reduced mobility who use any public transport system are most affected by rainfall. This may stimulate the development of scientific research and resource usage to understand the local mobility issues in developing countries. Thus, our findings contribute to the creation of a more sustainable and egalitarian city for PWD and elderly people. This is a novel use of the $\rho DCCA$ cross-correlation coefficient as an index of sustainable mobility, which can provide parameters for future studies in this field. In summary, this simple methodology seems to be useful for identifying public transportation routes and stations for which access must be improved.

## Methods

### Data

The data were obtained from the public bus transport institution, the City Hall of São Paulo, Municipal Department of Mobility and Transport, which provides open data on electronic tickets for all bus lines that circulate in the city. The city has about 1336 bus lines^67^. These data are organized in files for each day of the year. In this study, we analyzed the years 2015, 2016, and 2017, so we obtained 1096 files, considering 2016 as a leap year^68^.

**Figure 4:**
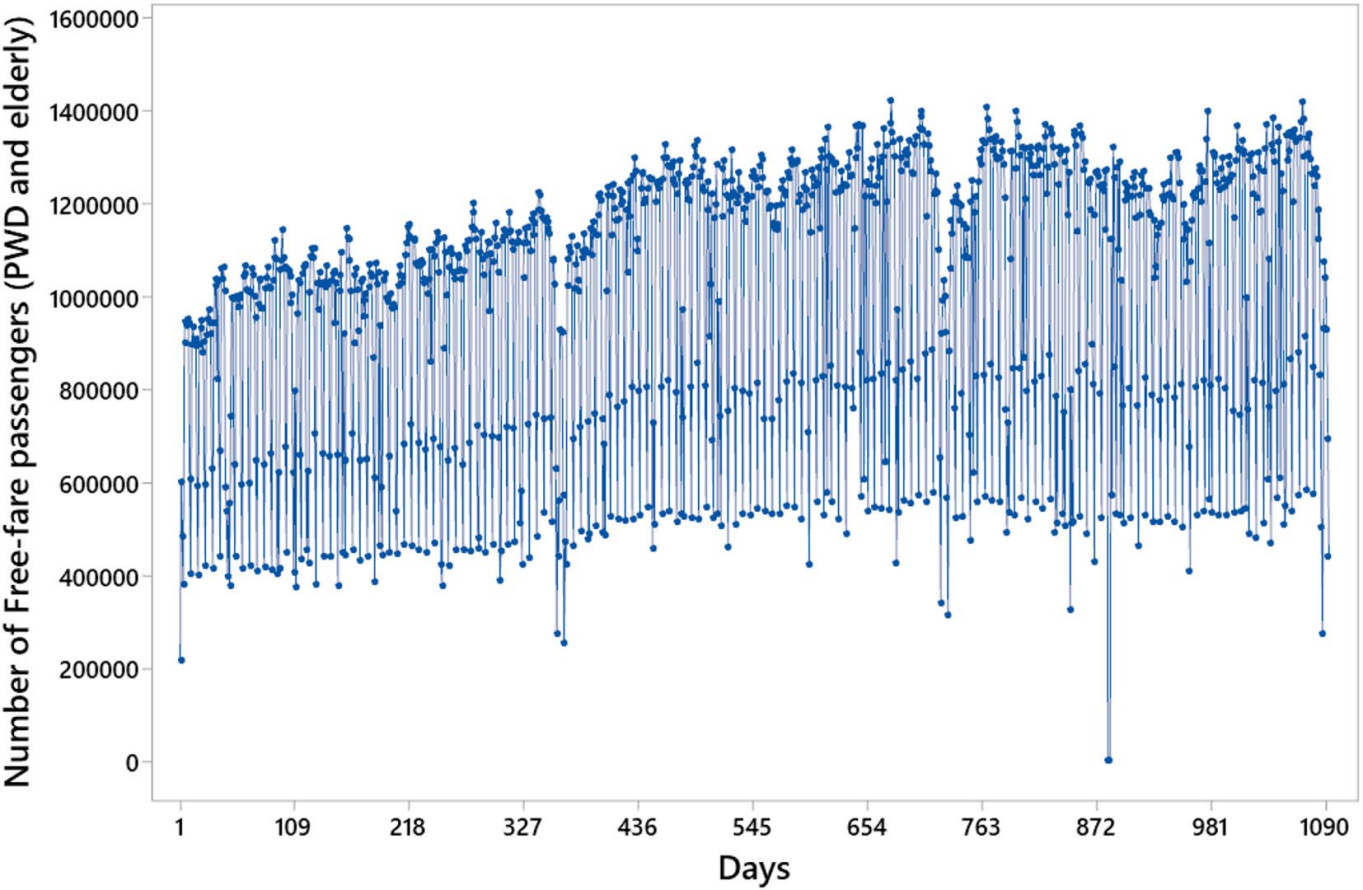
Original series with the number of PWD and elderly passengers riding the public bus transport system of São Paulo, Brazil, from January 2015 to December 2017 (daily values).

These files provide information about concession, area, company, and bus lines in circulation. Each file contains data on paying passengers in columns separated into passengers paying with cash, with common electronic tickets, with student electronic tickets, with transportation voucher electronic tickets (provided by the employer), and with metro-train connecting tickets from São Paulo Metropolitan Train Company (Companhia Paulista de Trens Metropolitanos (CPTM)). Lastly, all columns are summed into a single column^68^. Two other columns found in the file consisted of free-fare student passengers and free-fare PWD and elderly passengers. The latter was the focus of the study; therefore, only the column containing free-fare passenger data for PWD and elderly people was used for the exploratory analysis, as shown in Fig. 4.

The second set of data was obtained from the National Institute of Meteorology (Instituto Nacional de Meteorologia (INMET)) Mirante de Santana Meteorological Station in São Paulo (OMM: 83781), –23.5° latitude and 46.61° longitude^69^, which also provides daily open data, such as rainfall (mm), minimum and maximum temperature (°C), insolation (h), Piche evaporation (mm), mean compensated temperature (°C), mean relative humidity (%), and mean wind speed (mps).

**Figure 5:**
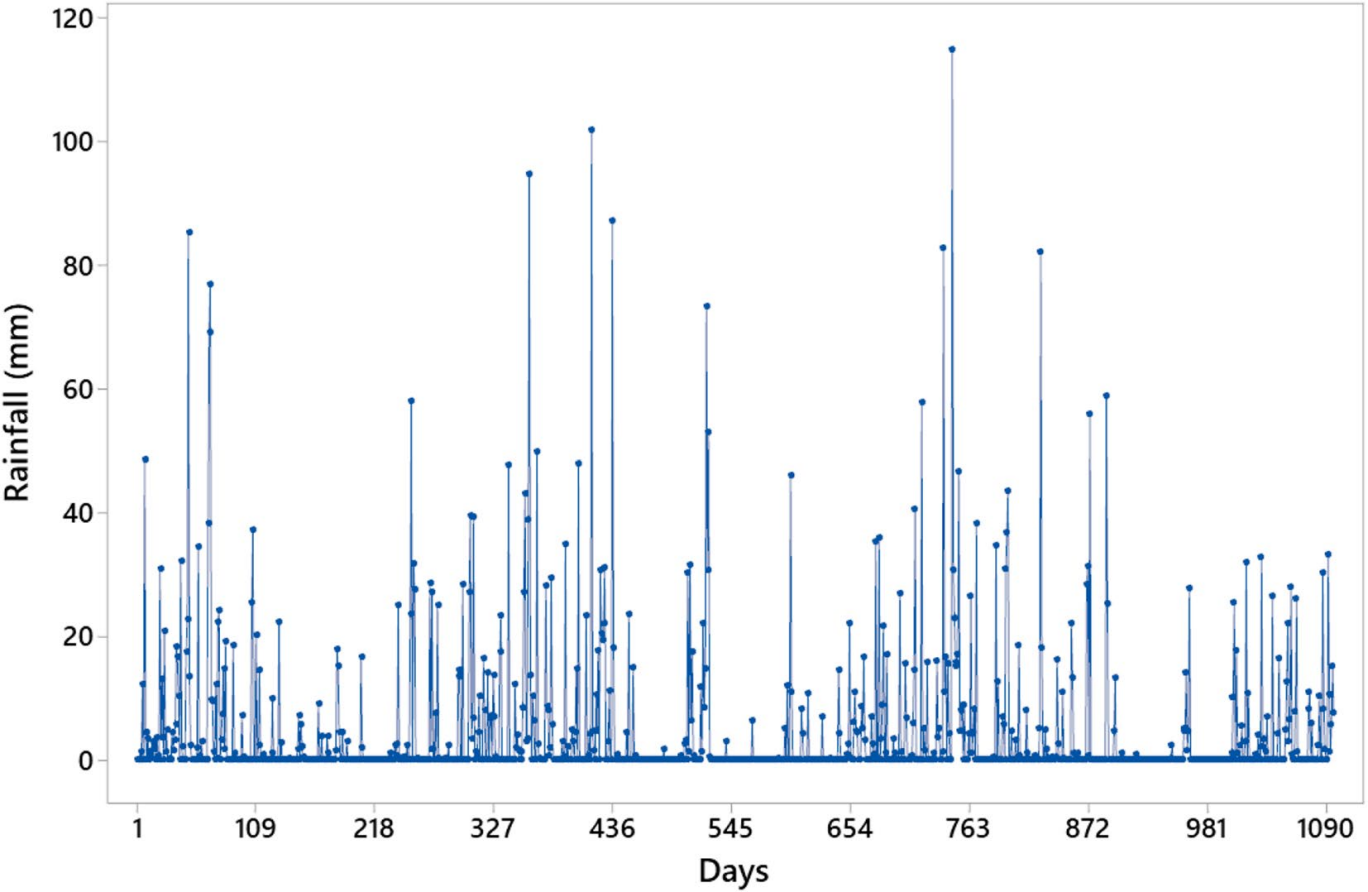
Original series with rainfall values in mm for the city of São Paulo, Brazil, from January 2015 to December 2017 (daily values).

The rainfall (mm) plotted in Fig. 5 expresses the daily amount of rain in the city. We considered the bus lines in regions close to the weather station. Therefore, the bus lines that pass through Santana station were selected for the study.

Thus, data from 30 bus lines leaving Santana station were selected and analyzed. Of these 30 lines, 10 had other regions of the city as a final destination, which were not in the north zone (for example, in the east, west, or south zones). There were 20 lines with final destinations in the north region of the city. The north zone encompasses a large portion of the city, and is the second largest geographic region in São Paulo, with an area of 152 km^2^ and a population of 1,181,582 of the 11,253,503 population of the entire city^70^.

### $\rho DCCA$

The DCCA cross-correlation coefficient ($\rho DCCA$) method^71^ involves a dimensionless coefficient that was used to quantify the level of cross-correlation between these two non-stationary time series. This method is based on the other two others detrended fluctuation analyses (DFAs)^72^ and the detrended cross-correlation analysis (DCCA)^73^ methods.

The DCCA cross-correlation coefficient ($\rho DCCA$) method estimate the existence of long-range cross-correlations between time series at different timescales *n*, which in this study were analyzed the series with the number of PWD and elderly passengers riding the public bus transport system of São Paulo and the series with rainfall values in mm for the same city in the period from 2015 to 2017 considering every day, except weekends and holidays.

The lack of stationarity is an attribute of the stochastic process, where time series that follow these characteristics require a linear transformation in the data sets or the use of nonparametric statistics should be considered. The cross-correlation between time series takes multi-timescale features, presenting differences in results throughout the timescales^74^, that is, being able to visually present a behavior trend from a certain time scale. To verify the associations between the means of precipitation and public bus transport passengers in São Paulo, we adopted $\rho DCCA$, which is a proposition that was implemented by^75^.

In recent studies, DCCA and Pearson methods have been compared^74^. It was found that (1) DCCA-related methods can quantify scale-dependent correlations, but the Pearson method cannot; (2) the correlation features from DCCA-related methods are robust to noise contamination; however, the results from the Pearson method are sensitive to noise; (3) the scale-dependent correlation results from DCCA-related methods are robust to the amplitude ratio between slow and fast components, whereas the Pearson method may be sensitive to the amplitude ratio. All these features indicate that DCCA-related methods provide some advantages in correctly quantifying scale-dependent correlations, which result from different physical processes.

The $\rho DCCA$ method has been applied in different fields such as climatological data^76^, economics^77, 78^, financial markets^79–83^, health^84^, and environment^76, 85^.

The $\rho _{DCCA}$ is determined by Eq. (1):

1 $$\begin{aligned} \rho _{DCCA}(s) = \frac{F^{2}\,_{xy}\left( s\right) }{F_{xx}\left( s\right) F_{yy}\left( s\right) }, \end{aligned}$$

where $F^{2}\,_{xy}\left( s\right)$ is the correlation function determined by the method of^73^; $F_{xx}\left( s\right)$ and $F_{yy}\left( s\right)$ are the auto-correlation functions determined by the method of^86^.

The level of the cross-correlation coefficient $\rho _{DCCA}$ has a range of [− 1, 1], where 1 equals a perfect cross-correlation and − 1 is a perfect anti-cross-correlation. A 0 value represents a non-cross-correlated condition^87^.
